# Predictive factors for recording the death of women of childbearing age in the Hospital Information System (SIH/SUS), Brazil, 2012–2020

**DOI:** 10.1590/1980-549720240051

**Published:** 2024-11-22

**Authors:** Juliana Alves Marques, Rosa Maria Soares Madeira Domingues, Marcos Augusto Bastos Dias, Claudia Medina Coeli, Rejane Sobrinho Pinheiro, Valeria Saraceni

**Affiliations:** I Pontifícia Universidade Católica do Paraná, Postgraduate Program in Urban Management – Curitiba (PR), Brazil.; II Fundação Oswaldo Cruz, Evandro Chagas National Institute of Infectious Diseases – Rio de Janeiro (RJ), Brazil.; III Fundação Oswaldo Cruz, Fernandes Figueira National Institute of Women’s, Children’s and Adolescents’ Health – Rio de Janeiro (RJ), Brazil.; IV Universidade Federal do Rio de Janeiro, Institute of Collective Health Studies – Rio de Janeiro (RJ), Brazil.; V Municipal Health Department of Rio de Janeiro – Rio de Janeiro (RJ), Brazil.

**Keywords:** Statistical databases, Information systems, Death, Medical assistance

## Abstract

**Objective:**

To estimate the death registration coverage of women of childbearing age (WCA) in the Hospital Information System (SIH), according to the hospital of occurrence and to verify the predictors associated with coverage.

**Methods:**

Descriptive ecological study with public data from SIH, Mortality Information System (SIM) and National Registry of Health Establishments (CNES), 2012–2020. Deaths in WCA hospitalizations in SIH were compared to those in SIM. Coverage was calculated by the proportion of deaths in SIH in relation to SIM. Supervised classification models — decision tree and random forest — were used to identify hospital characteristics related to coverage.

**Results:**

WCA death registration coverage was estimated at 78.0 and 71.8% after excluding hospitals with >100% coverage. Lower coverage was observed in the North region (67.7%) and higher in the South (76.9%). There was an increase in coverage from 69.0% to 74.4% in the period examined. The main factors predicting coverage were urgency/emergency facility, administrative management level, hospital complexity, proportion of adult beds covered by SUS and teaching activity, with lower coverage in those with an urgency/emergency facility and greater coverage in those of higher complexity, in federal hospitals, those with teaching activity and higher proportion of adult beds covered by SUS. Flaws in the CNES registration were identified in SIM.

**Conclusion:**

The coverage of WCA death registration in SIH in the country is high and growing. Regional differences reinforce the need for strategies to improve the quality of information systems.

## INTRODUCTION

The Hospital Information System of the Unified Health System (SIH/SUS), implemented in 1991 with the aim of reimbursing expenses for hospitalizations in public hospitals and hospitals affiliated with SUS^1^, has been used to analyze in-hospital mortality in several areas of care and for different purposes, including temporal analyses^2^, comparison of mortality rates between services^3^, assessment of underreporting of diseases subject to compulsory notification^4^, and study of specific causes^5,6^. Particularly in the area of maternal and perinatal health, studies have assessed fetal and neonatal mortality^7,8^ and in-hospital maternal mortality^9,10^.

However, for the SIH/SUS to be used to study in-hospital mortality, it is necessary to assess the coverage of death registration in this system^11^. Two previous studies assessed the coverage of death registration in SIH/SUS^12,13^. Amaral^12^, in 1998, compared the registration of deaths in the Mortality Information System (SIM) and in SIH/SUS in Brazilian capitals. The death records were compared according to the place of occurrence, and deaths that had received medical care were selected from SIM. Machado et al.^13^ compared hospital deaths reported in SIM with those reported as a result of SUS and non-SUS hospitalizations in 2009.

No studies were identified that evaluated the coverage of maternal death registration in SIH/SUS, but one study compared the maternal mortality ratio (MMR) estimated with data from SIM and SIH/SUS^10^. The authors of this study did not find significant differences between MMR estimated by the two systems and concluded that it is possible to use the SIH/SUS as a complement for studies of maternal mortality and morbidity^10^, with one advantage of SIH/SUS being the faster availability of death data compared to SIM^7^.

However, some particularities of maternal deaths make it difficult to compare records of these deaths in SIM and SIH/SUS. Maternal deaths have a specific definition, based on the time of death—during pregnancy, childbirth, or up to 42 days after the end of pregnancy—and on the underlying causes of death according to the International Classification of Diseases (ICD)^14^. A meta-analysis including 29 studies conducted in several countries estimated underreporting of maternal deaths at 39% (95%CI 30-48%), due to incomplete data or classification errors^15^. In Brazil, maternal deaths are subject to mandatory investigation, aiming to reduce underreporting and improve the quality of information, with investigation coverage exceeding 90%. In SIH/SUS, however, it is not possible to adequately classify maternal deaths, since the date of end of pregnancy is not available to identify deaths occurring up to 42 days after the end of pregnancy. Furthermore, the causes of death are not always filled in on the Hospital Admission Authorization (AIH), and these are not subject to investigation, with the cause recorded by the hospital team being available, subject to classification errors^16^.

The identification of obstetric hospitalizations is not simple, as there is no specific field that allows their identification. Authors have used definitions based on ICD, procedures and type of billing^10,17,18,19,20^, resulting in different classifications. Furthermore, not every hospitalization of a pregnant woman is an obstetric hospitalization, as it can be caused by chronic diseases and non-obstetric causes, such as external causes.

For all these reasons, we consider that the evaluation of the death record of women of childbearing age (WCA) is more appropriate for evaluating the coverage of death records in SIH/SUS than that of maternal deaths. WCA deaths only require the appropriate registration of sex and age in the AIH, a form that must be completed for all publicly funded hospitalizations, regardless of the reason for hospitalization and cause and time of death. Furthermore, maternal death surveillance begins with the investigation of WCA deaths, implemented in the country in 2008^21^, with the aim of identifying unreported maternal deaths and reducing underreporting of maternal deaths.

With the aim of contributing to the use of SIH/SUS for the study of in-hospital maternal mortality, this study aimed to evaluate the national coverage of WCA death registration in SIH/SUS, according to the establishment of occurrence of hospitalization, and to verify the institutional predictors of adequate coverage.

## METHODS

### Study design

Ecological study using data from SIH/SUS, SIM and the National Registry of Health Establishments (CNES), publicly available, from 2012-2020.

### Data source and processing

The SIM and SIH/SUS data were collected by the microdatasus package of the R software^22^ (https://github.com/rfsaldanha/microdatasus), and the CNES data were collected by downloading the files from the Data Science Platform Applied to Health (PCDaS/Fiocruz) (https://pcdas.icict.fiocruz.br/).

In SIM, all death certificates (DCs) of WCA (10 to 49 years old) that occurred in the period 2012–2020 in public or private hospitals with adult beds covered by SUS were selected. Data from the CNES were used to identify these hospitals. DCs without CNES registration or with invalid CNES were excluded from the analysis.

In SIH/SUS, all AIH for women aged 10 to 49 years old issued between 2012 and 2020 were selected. AIH with an unfilled or invalid CNES number were excluded from the analysis.

In the CNES, all establishments that had adult beds covered by SUS in the period 2012–2020 were identified, and an annual database was prepared for each establishment, containing: CNES number; administrative management (categories municipal, state, federal, private, not reported); number of adult beds (total and those covered by SUS); hospital complexity (variable “NIV_HIER”, with category “5” being low complexity, “6” and “7” medium complexity, “8” high complexity and “9” not reported); teaching activity (variable “ACTIVITY”, with category “1” being a university hospital, “2” and “3” other types of teaching, “4” without teaching activity and “99” not reported); existence of emergency/urgency services (variable URGEMERG, with category “1” indicating an emergency/urgency facility and “0” indicating no). The choice of these variables was based on expert evaluation and available studies on the subject^5,10,12,13^. Since the CNES database is monthly, the average was used for quantitative variables and the mode for qualitative variables to construct the annual database. The same criterion was used for the analyses related to the period 2012–2020. Establishments that did not have “adult beds covered by SUS” throughout the period were excluded from the analysis.

### Analysis of death coverage of women of childbearing age in the Hospital Information System/Unified Health System

The coverage of WCA death records in the SIH/SUS by health facility was calculated using the number of WCA AIH with discharge type “death” as the numerator, and the number of WCA deaths recorded in SIM in the same facility and year of occurrence as the denominator, multiplied by 100. In the AIH, hospital discharges due to death were identified in the variable “charge (reason for closure)”, response options 4.1, 4.2, 4.3, 6.5, 6.6 and 6.7. The identification of records in the two databases used the CNES code and the year as the key.

Subsequently, the facilities were grouped by federative unit (FU), and the coverage was calculated by FU, macro-region of the country and country. Total coverage was calculated, considering all records, and coverage after excluding hospitals with death records in SIH/SUS greater than 100%, for having been considered values resulting from registration errors.

Finally, the temporal trend of death coverage was evaluated, after excluding hospitals with coverage greater than 100%, for the country and the macro-region using Spearman’s correlation^23^.

### Analysis of predictors of death coverage

Initially, an exploratory analysis was performed to determine the coverage of death registration according to hospital characteristics:

1. Administrative management (municipal, state, federal, private, no information);
2. Proportion of adult beds covered by SUS (ratio between the number of adult beds covered by SUS and the total number of adult beds in the hospital unit, categorized into quartiles);
3. Size of the hospital (up to 50 beds “small”, 51 to 150 “medium”, 150 to 500 “large” and more than 500 beds “extra capacity”);
4. Hospital complexity (low, medium or high);
5. Teaching activity (university hospital, other type of teaching, no teaching activity, not informed); and
6. Emergency/urgency facility (yes or no).

Subsequently, supervised classification statistical models — decision tree and random forest — were used to identify the most relevant factors for predicting coverage. In both models, records with “no information” for the variables evaluated were excluded, resulting in the exclusion of 3.7% of the total records. For the decision tree, training data were used in a 60/40 ratio, i.e., 60% for training and 40% for testing in the database, with a record coverage equal to or greater than 90% being used as the adequacy parameter^24^. The order of importance of the hospital profile variables was analyzed using a random forest model, considering the same parameters as the decision tree model. The Mean Decrease Gini metric was also used, which assesses the importance of the variables in this model (calculated based on the average reduction in Gini impurity that each variable contributes across all trees in the forest). Gini impurity is a measure of the homogeneity of the data in a node of the tree. The lower the impurity, the greater the tendency for a node to contain mainly samples from a single class, while an impure node contains samples from several different classes. In both models, the positive class, that is, the one that represents the category to be identified or predicted, was defined as “inadequate” (record coverage less than 90%).

For the analysis, the R software in Rstudio^25^ was used. This study used only unidentified public access databases and was exempt from ethical assessment.

## RESULTS

**Figure 1:**
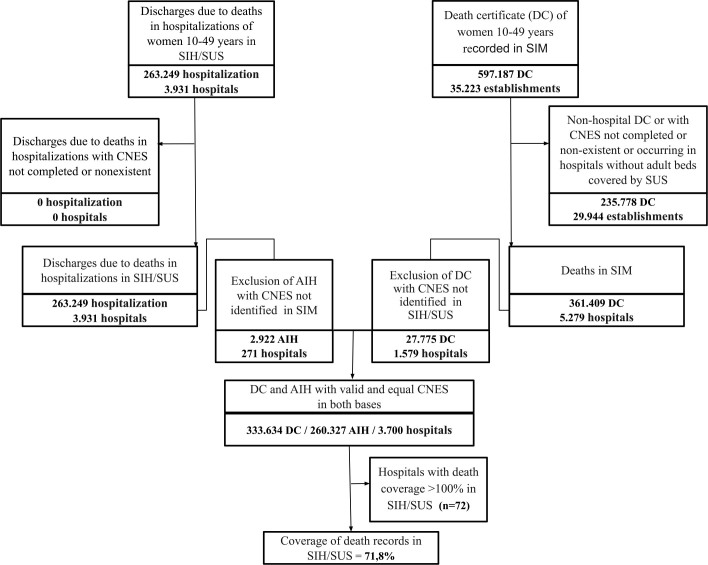
Flowchart of the analysis of the coverage of deaths of women of childbearing age in the Hospital Information System of the Unified Health System. Brazil; 2012–2020.

In the period 2012-2020, 597,187 WCA deaths were identified in SIM. After excluding the DCs of non-hospital deaths, with unfilled out or nonexistent CNES, or that occurred in hospitals without adult beds covered by SUS (total 235,778), 361,409 DCs of WCA deaths that occurred in hospitals with adult beds covered by SUS were identified. In the same period, 263,249 deaths in WCA hospitalizations were identified in SIH/SUS, all with an existing CNES record. In both databases, 333,634 DCs and 260,327 AIH with the same CNES number were identified, resulting in a national death registration coverage of 78%, with a reduction to 71.8% after excluding hospitals with coverage greater than 100% (Figure 1). DCs with unfilled out or invalid CNES excluded from the SIM database corresponded to 3.7% of deaths in hospitals, with the largest exclusions observed in Goiás, Amapá, Maranhão and Tocantins. DC and AIH excluded because of lack of correspondence in the CNES resulted in a loss of 7.7% of WCA deaths recorded in SIM and 1.1% of those recorded in SIH. The states with the greatest loss of deaths in SIM due to CNES without correspondence were Goiás, Maranhão, Rio Grande do Norte, Piauí and Roraima.

SIH/SUS: Hospital Information System of the Unified Health System; DC: Death certificate; SIM: Mortality Information System; CNES: National Health Establishment Registry; AIH: Hospital Admission Authorization.

The only FU with coverage greater than 100% was the Federal District. After excluding hospitals with coverage greater than 100%, the coverage ranged from 67.7% in the North region to 76.9% in the South region. The states with the highest coverage were Pernambuco (79.2%), Paraná (78.6%), Santa Catarina (77.8%) and Tocantins (77.6%), while those with the lowest coverage were Amapá (57.6%), Amazonas (60.9%), Alagoas (64.3%) and Paraíba (64.7%) (Table 1). The South region had the highest coverage throughout the period (76.9%, 95%CI 75.8–78.0), and the North region had the lowest (67.6%, 95%CI 65.0–70.2).

**Table 1:** Assessment of coverage of deaths of women of childbearing age by macro-region and federative unit in the Hospital Information System of the Unified Health System. Brazil; 2012–2020.

FU/HIS	SIM			SIH	SIM/SIH comparison					
	Total deaths of women aged 10-49 years (WCA)	Total deaths of WCA in hospitals with adult beds covered by SUS with a completed and valid CNES record		WCA deaths with completed and valid CNES	DC and AIH with valid and equal CNES in both databases				Coverage of WCA deaths	Coverage in ≤100%*
	n	n	(%)	N	SIM		SIH		%	%
					N	%†	n	%‡		
North	51,134	30,659	60.0	20,604	27,902	91.0	20,304	98.5	72.8	67.7
AC	2,394	1,533	64.0	1,054	1,449	94.5	1,047	99.3	72.3	69.3
AM	11,440	7,182	62.8	4,206	6,454	89.9	4,151	98.7	64.3	60.9
AP	2,260	1,560	69.0	929	1,524	97.7	921	99.1	60.4	57.6
PA	23,974	13,862	57.8	9,467	12,522	90.3	9,303	98.3	74.3	69.7
RO	4,898	2,998	61.2	2,095	2,628	87.7	2,084	99.5	79.3	68.6
RR	1,757	1,087	61.9	846	1,047	96.3	845	99.9	80.7	76.9
TO	4,411	2,437	55.2	2,007	2,278	93.5	1,953	97.3	85.7	77.6
Northeast	167,112	101,342	60.6	70,734	90,810	89.6	69,622	98.4	76.7	70.5
AL	10,838	6,986	64.5	4,503	6,658	95.3	4,485	99.6	67.4	64.3
BA	45,762	27,169	59.4	18,796	24,655	90.7	18,364	97.7	74.5	70.6
CE	24,951	15,127	60.6	10,021	13,519	89.4	9,878	98.6	73.1	69.6
MA	19,807	10,991	55.5	7,608	9,488	86.3	7,535	99.0	79.4	72.0
PB	11,651	7,575	65.0	4,766	6,868	90.7	4,755	99.8	69.2	64.7
PE	29,058	18,004	62.0	14,197	16,054	89.2	13,950	98.3	86.9	79.2
PI	9,371	5,717	61.0	4,530	5,016	87.7	4,512	99.6	90.0	65.4
RN	9,045	5,561	61.5	3,587	4,827	86.8	3,484	97.1	72.2	68.4
SE	6,629	4,212	63.5	2,726	3,725	88.4	2,659	97.5	71.4	64.6
C-West	46,840	26,188	55.9	19,506	23,266	88.8	19,382	99.4	83.3	72.5
DF	7,018	4,236	60.4	4,579	3,937	92.9	4,561	99.6	115.8	77.5
GO	20,830	10,431	50.1	6,824	8,936	85.7	6,772	99.2	75.8	71.0
MS	8,425	4,988	59.2	3,543	4,562	91.5	3,528	99.6	77.3	73.3
MT	10,567	6,533	61.8	4,560	5,831	89.3	4,521	99.1	77.5	72.7
Southeast	251,355	150,144	59.7	111,511	141,734	94.4	110,580	99.2	78.0	71.5
ES	11,434	6,937	60.7	4,598	6,436	92.8	4,566	99.3	70.9	67.7
MG	59,142	35,165	59.5	26,598	32,755	93.1	26,218	98.6	80.0	75.9
RJ	60,313	34,218	56.7	23,508	31,633	92.4	23,371	99.4	73.9	65.6
SP	120,466	73,824	61.3	58,807	70,910	96.1	56,425	99.3	79.6	72.7
South	80,746	53,076	65.7	40,894	49,92	94.1	40,439	98.9	81.0	76.9
PR	31,370	19,718	62.9	15,707	18,655	94.6	15,585	99.2	83.5	78.6
RS	31,692	21,716	68.5	16,124	20,220	93.1	15,891	98.6	78.6	74.8
SC	17,684	11,642	65.8	9,063	11,047	94.9	8,963	98.9	81.1	77.8
Brazil	597,187	361,409	60.5	263,249	333,634	92.3	260,327	98.9	78.0	71.8

FU: Federative unit; HIS: Health Information System; SIM: Mortality Information System; SIH: Hospital Information System; WCA: Women of childbearing age (10 to 49 years); SUS: Unified Health System; CNES: National Registry of Health Establishments; DC: Death certificate; AIH: Hospital Admission Authorization; C-West: Central-West.

*Coverage after exclusion of hospitals with coverage >100%;

†Proportion of DC with completed and valid CNES identified in SIH/SUS;

‡Proportion of AIH with completed and valid CNES identified in SIM.

**Figure 2:**
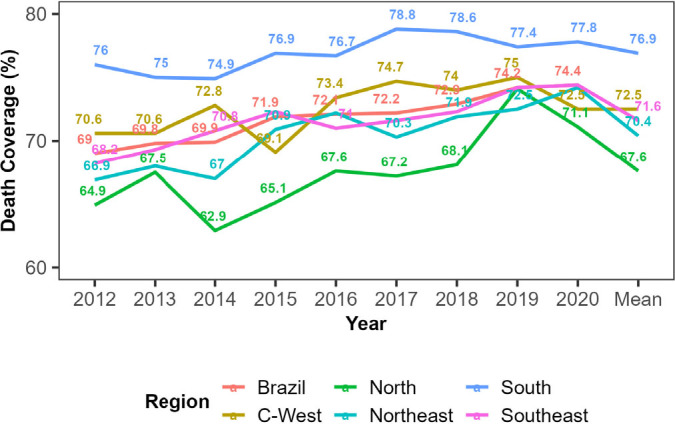
Time series of coverage of deaths of women of childbearing age in the Hospital Information System of the Unified Health System by macro-region of the country. Brazil; 2012–2020.

An increase in national coverage was observed during the period, increasing from 69% in 2012 to 74.4% in 2020. The trend of increasing coverage of WCA deaths in the period analyzed was significant in all macro-regions, except the Central-West (Figure 2).

In the descriptive analysis of the characteristics of the establishment associated with the coverage of WCA death registration at the Brazilian level, we found that the highest values were observed in larger establishments (84.4% extra capacity, 74.8% for large-scale, 69.2% for medium-scale and 54.9% for small-scale), at the federal level (81.6%, followed by the private level, with 73.5%; state, with 72.2%; and municipal, with 65.5%), with a higher proportion of SUS adult beds (72.3% third quartile [cut-off 91.5% of the proportion], 74.2% coverage second quartile [cut-off 84.4% of the proportion] and 68.1% first quartile [cut-off 70.9% of the proportion]), with teaching activity (university, 80.5%; other types of teaching 76.9% and without teaching activity, 68.3%), of greater complexity (high 74.2%, medium 64.4%, low 59.8%) and without emergency/urgency facilities (78.2% without facilities and 71.6% with facilities).

**Figure 3:**
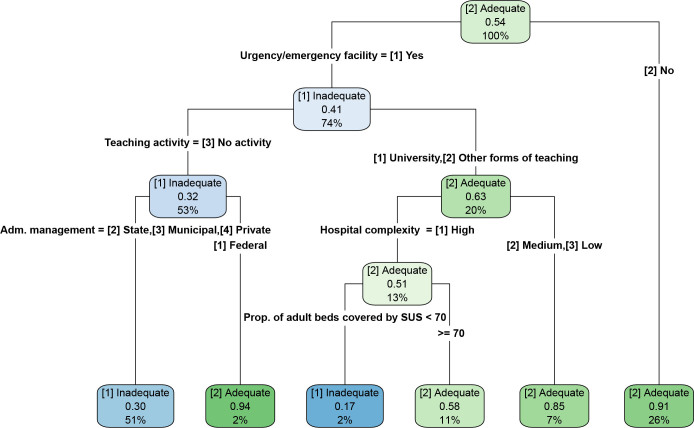
Decision tree for coverage of death registration of women of childbearing age in SIH/SUS. Brazil, 2012–2020.

**Figure 4:**
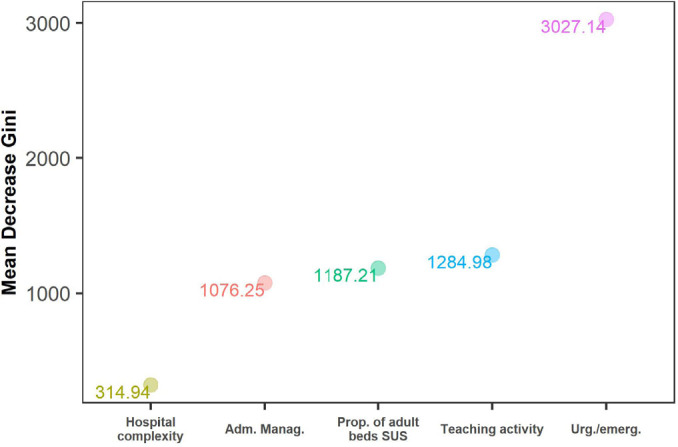
Random Forest of Death Record Coverage of Women of Childbearing Age in the Hospital Information System of the Unified Health System. Brazil; 2012–2020.

In the decision tree, the variables “emergency/urgency facilities” and “teaching activity” appeared in the first nodes of the tree. The following were classified as having the highest probability of inadequate coverage: hospitals with emergency/urgency facilities, without teaching activities at the state, municipal and private levels (51% of the total base); and hospitals with emergency/urgency facilities, with teaching activities, of high complexity and with a proportion of SUS adult beds <70% (2% of the total base) (Figure 3). In the random forest, the main predictors of WCA death coverage were having an emergency/urgency facility, teaching activity, proportion of adult beds covered by SUS, administrative management and level of complexity (Figure 4).

SIH/SUS: Hospital Information System of the Unified Health System; Adm. Management: administrative management (state, municipal, private, federal); Prop. of adult beds covered by SUS: proportion of adult beds covered by SUS in relation to the total number of adult beds available.

Mean Decrease Gini: parameter used to assess the importance of variables in the model; hospital complexity: level of hospital complexity (low, medium, high); Adm. Manag.: administrative management (municipal, state, federal, private, not indicated); prop. of adult beds SUS: proportion of adult beds covered by SUS in relation to the total number of adult beds available; teaching activity: university, other types of teaching, no teaching activity; Urg/emerg: existence of urgency/emergency physical facility (yes/no).

The two models showed similar accuracies — around 66% —, but with slightly different performances regarding predictions of establishments with or without adequate coverage. The decision tree showed a higher positive predictive value (82.4 vs. 72.4%) and specificity (37.9 vs. 21.9%), while the random forest presented greater sensitivity (85.3 vs. 72.8%) and negative predictive value (38.2 vs. 25.7%).

## DISCUSSION

The results found in this study show a national coverage of WCA death registration in SIH/SUS of 71.8%, with an increase in the period 2012–2020. The highest coverage was observed in more complex services, at the federal level, with a higher proportion of adult beds covered by SUS and in teaching hospitals; the lowest coverage was observed in hospitals with emergency/urgent care facilities.

Comparison of our results with previous studies conducted in the 1990s and 2000s suggests an increase in death registration coverage, but these data should be analyzed with caution, because of differences in the methods used. Amaral^12^, in a study conducted in 1998, estimated that death registration coverage in the female population was 52.4%, with decreasing coverage according to age group: 89.3% in the population aged 5–14 years, 80.8% in the population aged 15–24 years, 71% in the population aged 25–44 years, and 62.5% in the population aged 45–54 years. In this study, deaths from SIH/SUS were compared to deaths in SIM that had a medical care record, with no information regarding the service, whether public or private. This method of calculation may have underestimated the coverage of SIH/SUS records, since the total number of deaths with medical care recorded in SIM included deaths in public and private services, while deaths recorded in SIH/SUS refer only to hospitalizations in public services.

Machado et al.^13^ estimated that the coverage of deaths recorded in hospitalizations under SUS and non-SUS was 56.8% in 2009, while the coverage in public services was 57.8%. Non-SUS deaths were estimated on the basis of the number of deaths of health plan beneficiaries disclosed by the ANS on the internet, while SUS deaths were estimated by the difference between the total number of deaths in the SIM and the non-SUS deaths. Failures in the registration and communication of deaths in private services may have overestimated the number of deaths in public services in SIM and resulted in lower coverage of records in this sector.

Similar to what was observed in this study, lower coverage was observed by Machado et al.^13^ in the North region and higher coverage in the South region. More complex, larger services, with teaching activities and with a higher proportion of adult beds covered by the SUS were those that showed the highest coverage of recorded deaths, and it is likely that the distribution of these services is uneven in the country, affecting coverage by region. However, it is worth noting the significant increase in death registration coverage in the North region during the period evaluated, with a reduction in the difference in relation to other regions.

Greater coverage of death registration in services with a higher proportion of SUS beds would be an expected result, since not all deaths occurring in services affiliated with SUS are publicly funded and are registered in SIM but not in SIH/SUS. This was one of the explanations for the lower MMR estimated in SIH/SUS in relation to SIM in the study conducted by Ranzani et al.^10^. On the other hand, greater coverage in larger, more complex services with teaching activities may reflect a better structure and organization of the medical registration and documentation systems in establishments with these characteristics. Greater coverage of hospitalization registration in larger hospitals had already been reported by Machado et al.^13^, as well as better performance of hospital management in larger and more complex hospitals^26^.

The factor that most affected death registration coverage was the existence of an emergency/urgent care facility in the hospital. Lower death registration in hospitals with emergency/urgent care services had previously been reported in a study that assessed mortality from acute myocardial infarction^5^. Our hypothesis for this result is that in these hospitals, death certificates are issued for patients admitted to the emergency/urgent care unit who died, without the corresponding issuance of an AIH for these same individuals. Thus, deaths are registered in SIM, but not in SIH/SUS, resulting in lower coverage. The under-registration of WCA deaths in emergency/urgent care services may affect estimates of maternal deaths, especially those occurring in the postpartum period, due to complications not specific to pregnancy, such as infections.

The prediction models showed similar accuracy, differing in specific measures, with greater sensitivity and negative predictive value in the random forest. Random forests tend to generate better predictions than decision trees, but the decision tree results can be easier to implement by managers, since it generates cutoff points for variables (quantitative or qualitative), allowing the visualization of “classification rules” and their respective values. For example, in the tree presented, hospitals with emergency/urgency facilities; without teaching activities; and those from the state, municipal, and private administrative management showed the highest probability of inadequate coverage, representing 50% of the database, and should be the priority focus of improvement strategies.

This study has limitations related to the quality of data recording. DCs with missing or invalid CNES were excluded from the SIM, as well as DCs and AIH with CNES numbers without correspondence in SIH/SUS and SIM. Hospital units that displayed coverage greater than 100% were also excluded, because of the possibility of recording errors, since SIM shows coverage greater than 90% in the country, and SIH/SUS coverage greater than 100% would not be expected. These problems did not occur uniformly across the states and may have affected coverage by state, in addition to national coverage. CNES registration errors and the issuance of AIH for hospitalizations that were not performed are possible explanations for coverage in SIH/SUS exceeding 100%.

The frequencies of death registration in SIM and SIH/SUS were compared in each hospital unit, and it was not possible to state whether there was agreement between the deaths recorded. It is possible that there is under-registration of death information, since the outcome of hospitalization does not affect payment for procedures performed^13^. On the other hand, typing errors may result in over-registration of deaths, since there are no critical issues in the system and typing the ICD of the death is not mandatory in SIH/SUS. The causes of death recorded in SIH/SUS and their agreement with the causes recorded in SIM were also not evaluated.

Administrative databases are intended to pay for care provided, not to assess the quality of care, and may present errors in filling in relevant variables^27,28^. Studies conducted in other countries also point to weaknesses in the availability, accessibility and quality of information on deaths in hospital registration systems^29,30,31^ and the need for training in data processing, including storage and periodic review of medical records^30^.

In conclusion, SIH/SUS presented high coverage of WCA death registration throughout the country, being an important source of information for the surveillance of maternal morbidity and mortality in the country. Strategies should be adopted to improve the registration of data in the various hospital information systems, including deaths in SIH/SUS. Future studies should evaluate the agreement between the registration of deaths in SIM and SIH/SUS, as well as their causes.
