# Supplementary material for: Predictive factors for recording the death of women of childbearing age in the Hospital Information System (SIH/SUS), Brazil, 2012–2020
Source: Rev Bras Epidemiol. 2024 Nov 22;27:e240051. doi: 10.1590/1980-549720240051 (PMC11653955; doi:10.1590/1980-549720240051)
Supplement: Translation

# Translation: Fatores preditivos do registro de óbito de mulher em idade fértil no Sistema de Internação Hospitalar (SIH/SUS), Brasil, 2012–2020

**DOI:** 10.1590/1980-549720240051.2

**Authors:** Juliana Alves Marques, Rosa Maria Soares Madeira Domingues, Marcos Augusto Bastos Dias, Claudia Medina Coeli, Rejane Sobrinho Pinheiro, Valeria Saraceni

**Affiliations:** I Pontifícia Universidade Católica do Paraná, Postgraduate Program in Urban Management – Curitiba (PR), Brasil.; II Fundação Oswaldo Cruz, Evandro Chagas National Institute of Infectious Diseases – Rio de Janeiro (RJ), Brasil.; III Fundação Oswaldo Cruz, Fernandes Figueira National Institute of Women’s, Children’s and Adolescents’ Health – Rio de Janeiro (RJ), Brasil.; IV Universidade Federal do Rio de Janeiro, Institute of Collective Health Studies – Rio de Janeiro (RJ), Brasil.; V Secretaria Municipal de Saúde do Rio de Janeiro – Rio de Janeiro (RJ), Brasil.

**Keywords:** Bases de dados estatísticos, Sistemas de informação, Óbito, Assistência hospitalar

## Abstract

**Objetivo:**

Estimar a cobertura de registro de óbito de mulheres em idade fértil (MIF) no Sistema de Informação Hospitalar (SIH), segundo hospital de ocorrência, e verificar os preditores associados à cobertura.

**Métodos:**

Estudo ecológico descritivo com dados públicos do SIH, Sistema de Informação sobre Mortalidade (SIM) e Cadastro Nacional de Estabelecimentos de Saúde (CNES) no período 2012–2020. Óbitos em internações de MIF no SIH foram comparados aos do SIM. Calculou-se a cobertura pela proporção de óbitos no SIH em relação ao SIM. Modelos de classificação supervisionada — árvore de decisão e floresta aleatória — foram utilizados para identificação das características hospitalares relacionadas à cobertura.

**Resultados:**

A cobertura de registro de óbito de MIF foi estimada em 78%, e em 71,8% após exclusão de hospitais com cobertura >100%. Menor cobertura foi observada na região Norte (67,7%) e maior na Sul (76,9%). Houve aumento da cobertura — de 69% para 74,4% — no período. Os principais fatores preditivos da cobertura foram instalação física de urgência/emergência, esfera administrativa, nível de complexidade, proporção de leitos adultos conveniados ao SUS e atividade de ensino, com menor cobertura naqueles com instalação de urgência/emergência e maior cobertura naqueles com maior complexidade, da esfera federal, com atividade de ensino e maior proporção de leitos adultos conveniados ao SUS. Falhas no registro do CNES foram identificadas no SIM.

**Conclusão:**

A cobertura de registro de óbito de MIF no SIH no país é elevada e crescente. Diferenças regionais reforçam a necessidade de estratégias de melhoria da qualidade dos sistemas de informação.

## INTRODUÇÃO

O Sistema de Informação Hospitalar do Sistema Único de Saúde (SIH/SUS), implantado em 1991 com o objetivo de realizar o ressarcimento das despesas das internações ocorridas em hospitais públicos e conveniados ao SUS^1^, tem sido utilizado para a análise da mortalidade intra-hospitalar em diversas áreas assistenciais e com objetivos diversos, incluindo análises temporais^2^, comparação de taxas de mortalidade entre serviços^3^, avaliação de sub-registro de doenças de notificação compulsória^4^ e estudo de causas específicas^5,6^. Especialmente na área da saúde materna e perinatal, estudos avaliaram a mortalidade fetal e neonatal^7,8^ e a mortalidade materna intra-hospitalar^9,10^.

Entretanto, para que o SIH/SUS possa ser usado para o estudo da mortalidade intra-hospitalar, é necessário avaliar a cobertura do registro dos óbitos neste sistema^11^. Dois estudos anteriores avaliaram a cobertura de registro de óbitos no SIH/SUS^12,13^. Amaral^12^, em 1998, comparou o registro de óbitos no Sistema de Informação sobre Mortalidade (SIM) e no SIH/SUS em capitais brasileiras. Os registros de óbito foram comparados segundo local de ocorrência, sendo selecionados no SIM os óbitos que haviam recebido assistência médica. Já Machado et al.^13^ compararam os óbitos hospitalares informados no SIM com aqueles informados como resultado das internações SUS e não SUS em 2009.

Não foram identificados estudos que avaliaram a cobertura de registro de óbitos maternos no SIH/SUS, tendo um estudo comparado a razão de mortalidade materna (RMM) estimada com dados do SIM e do SIH/SUS^10^. Os autores desse estudo não encontraram diferenças significativas entre a RMM estimada pelos dois sistemas e concluíram ser possível utilizar o SIH/SUS de forma complementar para estudos de mortalidade e morbidade materna^10^, sendo uma das vantagens do SIH/SUS a disponibilidade mais rápida dos dados de óbito do que o SIM^7^.

Entretanto, algumas particularidades da morte materna dificultam a comparação dos registros desses óbitos no SIM e no SIH/SUS. Óbitos maternos apresentam uma definição específica, baseada no momento de ocorrência do óbito — durante a gestação, o parto ou até 42 dias após o término da gestação — e nas causas básicas do óbito segundo a Classificação Internacional de Doenças (CID)^14^. Uma metanálise incluindo 29 estudos realizados em vários países estimou sub-registro de óbitos maternos de 39% (IC95% 30-48%), por incompletude de dados ou erros de classificação^15^. No Brasil, os óbitos maternos são de investigação obrigatória, visando reduzir o sub-registro e melhorar a qualidade da informação, com cobertura de investigação superior a 90%. Já no SIH/SUS, não é possível classificar adequadamente os óbitos maternos, pois não está disponível a data do término da gestação, para identificação dos óbitos ocorridos até 42 dias após o término da gravidez. Além disso, as causas do óbito nem sempre estão preenchidas na Autorização da Internação Hospitalar (AIH), e estas não são objeto de investigação, estando disponível a causa registrada pela equipe do hospital, sujeita a erros de classificação^16^.

A própria identificação das internações obstétricas não é simples, pois não existe um campo específico que permita a sua identificação. Autores têm utilizado definições baseadas em CID, procedimentos e tipo de cobrança^10,17,18,19,20^, resultando em classificações diferentes. Além disso, nem toda internação de uma gestante é uma internação obstétrica, pois pode ser motivada por doenças crônicas e causas não obstétricas, por exemplo, causas externas.

Por todas essas razões, consideramos que a avaliação do registro de óbitos de mulheres em idade fértil (MIF) é mais adequada para a avaliação da cobertura de registros de óbitos no SIH/SUS do que o de óbitos maternos. Óbitos de MIF necessitam apenas do registro adequado de sexo e idade na AIH, formulário que deve ser preenchido para todas as internações com financiamento público, não dependendo do motivo de internação, causa e momento do óbito. Além disso, a vigilância dos óbitos maternos se inicia com a investigação de óbitos de MIF, implantada no país em 2008^21^, tendo por objetivo identificar óbitos maternos não declarados e reduzir o sub-registro de mortes maternas.

Com o intuito de contribuir para o uso do SIH/SUS para o estudo da mortalidade materna intra-hospitalar, este artigo tem por objetivo avaliar a cobertura nacional de registro de óbitos de MIF no SIH/SUS, segundo o estabelecimento de ocorrência da internação, e verificar os preditores institucionais da cobertura adequada.

## MÉTODOS

### Desenho do estudo

Estudo ecológico utilizando dados do SIH/SUS, do SIM e do Cadastro Nacional de Estabelecimentos de Saúde (CNES), disponíveis publicamente, no período 2012-2020.

### Fonte e processamento de dados

Os dados do SIM e do SIH/SUS foram captados pelo pacote microdatasus do *software* R^22^ (https://github.com/rfsaldanha/microdatasus), e os dados do CNES por *download* dos arquivos da Plataforma de Ciência de Dados Aplicadas à Saúde (PCDaS/Fiocruz) (https://pcdas.icict.fiocruz.br/).

No SIM foram selecionadas todas as Declarações de Óbito (DO) de MIF (10 a 49 anos) ocorridos no período 2012–2020 em hospitais públicos ou privados com leitos adultos conveniados ao SUS. Para a identificação desses hospitais foram utilizados dados do CNES. Declarações de óbito sem registro de CNES ou com CNES inválido foram excluídas da análise.

No SIH/SUS foram selecionadas todas as AIH de mulheres de 10 a 49 anos emitidas entre 2012 e 2020. AIH com número de CNES não preenchido ou inválido foram excluídas da análise.

No CNES foram identificados todos os estabelecimentos que dispunham de leitos adultos com convênio SUS no período 2012–2020, sendo preparada uma base anual para cada estabelecimento, contendo: número do CNES; esfera (categorias municipal, estadual, federal, privada, não informado); número de leitos adulto (total e conveniados ao SUS); complexidade do hospital (variável “NIV_HIER”, sendo a categoria “5” baixa complexidade, “6” e “7” média complexidade, “8” alta complexidade e “9” não informado); atividade de ensino (variável “ATIVIDAD”, sendo categoria “1” hospital universitário, “2” e “3” outras modalidades de ensino, “4” sem atividade de ensino e “99” não informado); existência de serviços de urgência/emergência (variável URGEMERG, sendo a categoria “1” indicativa de instalação física de urgência/emergência e “0” não). A escolha dessas variáveis foi baseada em avaliação de especialistas e estudos disponíveis sobre o tema^5,10,12,13^. Como a base do CNES é mensal, para a construção da base anual foi utilizada a média para as variáveis quantitativas, e a moda para a variáveis qualitativas. O mesmo critério foi utilizado para as análises referentes ao período 2012–2020. Estabelecimentos que durante todo o período não apresentaram leitos do tipo “adulto conveniado ao SUS” foram excluídos da análise.

### Análise da cobertura de óbito de mulheres em idade fértil no Sistema de Informação Hospitalar/Sistema Único de Saúde

A cobertura de registro de óbito de MIF no SIH/SUS por estabelecimento de saúde foi calculada utilizando como numerador o número de AIH de MIF com tipo de saída “óbito”, e como denominador o número de óbitos de MIF registrados no SIM no mesmo estabelecimento e ano de ocorrência, multiplicado por 100. Na AIH, as saídas por óbito foram identificadas na variável “cobrança (motivo de encerramento)”, opções de resposta 4.1, 4.2, 4.3, 6.5, 6.6 e 6.7. A identificação de registros nas duas bases utilizou como chave o código do CNES e o ano.

Posteriormente, os estabelecimentos foram agrupados por Unidade da Federação (UF), sendo calculada a cobertura por UF, por macrorregião do país e país. Foi calculada a cobertura total, considerando todos os registros, e a cobertura após exclusão dos hospitais com registro de óbitos no SIH/SUS superior a 100%, por terem sido considerados valores decorrentes de erro de registro.

Por fim, foi avaliada a tendência temporal da cobertura de óbitos, após exclusão dos hospitais com cobertura superior a 100%, para o país e a macrorregião utilizando o teste de correlação de Spearman^23^.

### Análise dos fatores preditores da cobertura de óbitos

Inicialmente, foi feita análise exploratória para verificar a cobertura de registro de óbitos segundo características hospitalares:

1. Esfera (municipal, estadual, federal, privado, sem informação);
2. Proporção de leitos adultos conveniados ao SUS (relação entre o número de leitos adultos conveniado ao SUS e o total de leitos adultos da unidade hospitalar, categorizada em quartis);
3. Porte do hospital (até 50 leitos “porte pequeno”, de 51 a 150 “médio porte”, de 150 a 500 “grande porte” e mais de 500 leitos “capacidade extra”);
4. Complexidade do hospital (baixa, média ou alta);
5. Atividade de ensino (hospital universitário, outra modalidade de ensino, sem atividade de ensino, não informado) e
6. Instalação física de urgência/emergência (sim ou não).

Posteriormente, foram utilizados modelos estatísticos de classificação supervisionada — árvore de decisão e floresta aleatória — para identificar os fatores mais relevantes para a predição da cobertura. Em ambos os modelos, foram excluídos os registros “sem informação” das variáveis avaliadas, resultando na exclusão de 3,7% do total de registros. Para a árvore de decisão, foram utilizados dados de treinamento na proporção de 60/40, ou seja, 60% para treinamento e 40% para teste na base, sendo utilizado como parâmetro de adequação a cobertura de registro igual ou superior a 90%^24^. Foi analisada, a partir de um modelo de floresta aleatória, a ordem de importância das variáveis do perfil hospitalar, considerando os mesmos parâmetros do modelo de árvore de decisão. Foi utilizada, ainda, a métrica de Mean Decrease Gini, que avalia a importância das variáveis desse modelo (calculado com base na redução média da impureza de Gini que cada variável contribui ao longo de todas as árvores da floresta). A impureza de Gini é uma medida da homogeneidade dos dados em um nó da árvore. Quanto menor a impureza, maior a tendência de um nó conter principalmente amostras de uma única classe, enquanto um nó impuro contém amostras de várias classes diferentes. Em ambos os modelos, a classe positiva, ou seja, aquela que representa a categoria que se quer identificar ou prever, foi definida como “inadequado” (cobertura de registro inferior a 90%).

Para análise, foi utilizado o *software* R em *Rstudio* ^25^. Este estudo utilizou apenas bases de acesso público não identificadas, estando isento de apreciação ética.

## RESULTADOS

No período 2012-2020, foram identificados 597.187 óbitos de MIF no SIM. Após exclusão de DO de óbitos não hospitalares, com CNES não preenchido ou inexistente, ou ocorridos em hospitais sem convênio SUS (total 235.778), foram identificadas 361.409 DO de óbitos de MIF ocorridos em hospitais com leito adulto conveniado ao SUS. No mesmo período, foram identificados 263.249 óbitos em internações de MIF no SIH/SUS, todos com registro de CNES existente. Foram identificadas, nas duas bases, 333.634 declarações de óbito e 260.327 AIH com mesmo número de CNES, resultando numa cobertura nacional de registro de óbito de 78%, com redução para 71,8% após exclusão de hospitais com cobertura superior a 100% (Figura 1).

**Figura 1:** Fluxograma da análise da cobertura de óbitos de mulher em idade fértil no Sistema de Informação Hospitalar do Sistema Único de Saúde. Brasil; 2012–2020.

SIH/SUS: Sistema de Informação Hospitalar do Sistema Único de Saúde; DO: Declaração de óbito; SIM: Sistema de Informação de Mortalidade; CNES: Cadastro Nacional de Estabelecimento de Saúde; AIH: Autorização da Internação Hospitalar.

As DO com CNES não preenchido ou inválido excluídas da base do SIM corresponderam a 3,7% dos óbitos em hospitais, sendo as maiores exclusões observadas em Goiás, Amapá, Maranhão e Tocantins. DO e AIH excluídas por falta de correspondência do CNES resultaram numa perda de 7,7% dos óbitos de MIF registrados no SIM e 1,1% dos registrados no SIH. Os estados com maior perda de óbitos no SIM por CNES sem correspondência foram Goiás, Maranhão, Rio Grande do Norte, Piauí e Roraima.

A única UF que apresentou cobertura superior a 100% foi o Distrito Federal. Após a exclusão dos hospitais com cobertura superior a 100%, esta variou de 67,7% na região Norte a 76,9% na região Sul. As UF com maior cobertura foram Pernambuco (79,2%), Paraná (78,6%), Santa Catarina (77,8%) e Tocantins (77,6%), enquanto as com menor cobertura foram Amapá (57,6%), Amazonas (60,9%), Alagoas (64,3%) e Paraíba (64,7%) (Tabela 1). A região Sul apresentou a maior cobertura em todo o período (76,9%, IC95% 75,8–78,0), e a região Norte a menor (67,6%, IC95% 65,0–70,2).

**Tabela 1:** Avaliação da cobertura de óbitos de mulher em idade fértil por macrorregião e Unidade da Federação no Sistema de Internação Hospitalar do Sistema Único de Saúde. Brasil; 2012–2020.

UF/SIS	SIM			SIH	Comparação SIM/SIH					
	Total de óbitos de mulheres de 10-49 anos (MIF)	Total de óbitos de MIF em hospitais com leito adulto conveniado ao SUS com registro de CNES preenchido e válido		Óbitos de MIF com CNES preenchido e válido	DO e AIH com CNES válido e igual nas duas bases				Cobertura de óbitos MIF	Cobertura em ≤100%*
	n	n	(%)	N	SIM		SIH		%	%
					n	%†	n	%‡		
Norte	51.134	30.659	60,0	20.604	27.902	91,0	20.304	98,5	72,8	67,7
AC	2.394	1.533	64,0	1.054	1.449	94,5	1.047	99,3	72,3	69,3
AM	11.440	7.182	62,8	4.206	6.454	89,9	4.151	98,7	64,3	60,9
AP	2.260	1.560	69,0	929	1.524	97,7	921	99,1	60,4	57,6
PA	23.974	13.862	57,8	9.467	12.522	90,3	9.303	98,3	74,3	69,7
RO	4.898	2.998	61,2	2.095	2.628	87,7	2.084	99,5	79,3	68,6
RR	1.757	1.087	61,9	846	1.047	96,3	845	99,9	80,7	76,9
TO	4.411	2.437	55,2	2.007	2.278	93,5	1.953	97,3	85,7	77,6
Nordeste	167.112	101.342	60,6	70.734	90.810	89,6	69.622	98,4	76,7	70,5
AL	10.838	6.986	64,5	4.503	6.658	95,3	4.485	99,6	67,4	64,3
BA	45.762	27.169	59,4	18.796	24.655	90,7	18.364	97,7	74,5	70,6
CE	24.951	15.127	60,6	10.021	13.519	89,4	9.878	98,6	73,1	69,6
MA	19.807	10.991	55,5	7.608	9.488	86,3	7.535	99,0	79,4	72,0
PB	11.651	7.575	65,0	4.766	6.868	90,7	4.755	99,8	69,2	64,7
PE	29.058	18.004	62,0	14.197	16.054	89,2	13.950	98,3	86,9	79,2
PI	9.371	5.717	61,0	4.530	5.016	87,7	4.512	99,6	90,0	65,4
RN	9.045	5.561	61,5	3.587	4.827	86,8	3.484	97,1	72,2	68,4
SE	6.629	4.212	63,5	2.726	3.725	88,4	2.659	97,5	71,4	64,6
C. Oeste	46.840	26.188	55,9	19.506	23.266	88,8	19.382	99,4	83,3	72,5
DF	7.018	4.236	60,4	4.579	3.937	92,9	4.561	99,6	115,8	77,5
GO	20.830	10.431	50,1	6.824	8.936	85,7	6.772	99,2	75,8	71,0
MS	8.425	4.988	59,2	3.543	4.562	91,5	3.528	99,6	77,3	73,3
MT	10.567	6.533	61,8	4.560	5.831	89,3	4.521	99,1	77,5	72,7
Sudeste	251.355	150.144	59,7	111.511	141.734	94,4	110.580	99,2	78,0	71,5
ES	11.434	6.937	60,7	4.598	6.436	92,8	4.566	99,3	70,9	67,7
MG	59.142	35.165	59,5	26.598	32.755	93,1	26.218	98,6	80,0	75,9
RJ	60.313	34.218	56,7	23.508	31.633	92,4	23.371	99,4	73,9	65,6
SP	120.466	73.824	61,3	58.807	70.910	96,1	56.425	99,3	79,6	72,7
Sul	80.746	53.076	65,7	40.894	49.92	94,1	40.439	98,9	81,0	76,9
PR	31.370	19.718	62,9	15.707	18.655	94,6	15.585	99,2	83,5	78,6
RS	31.692	21.716	68,5	16.124	20.220	93,1	15.891	98,6	78,6	74,8
SC	17.684	11.642	65,8	9.063	11.047	94,9	8.963	98,9	81,1	77,8
Brasil	597.187	361.409	60,5	263.249	333.634	92,3	260.327	98,9	78,0	71,8

UF: Unidade da Federação; SIS: Sistema de Informação em Saúde; SIM: Sistema de Informação de Mortalidade; SIH: Sistema de Informação Hospitalar; MIF: Mulher em idade fértil (10 a 49 anos); SUS: Sistema Único de Saúde; CNES: Cadastro Nacional de Estabelecimento de Saúde; DO: Declaração de óbito; AIH: Autorização da Internação Hospitalar; C. Oeste: Centro-Oeste.

*Cobertura após exclusão de hospitais com cobertura >100%;

†Proporção de DO com CNES preenchido e válido identificadas no SIH/SUS;

‡Proporção de AIH com CNES preenchido e válido identificadas no SIM.

Foi observado aumento da cobertura nacional no período, passando de 69% em 2012 a 74,4% em 2020. A tendência de aumento da cobertura de óbitos de MIF no período analisado foi significativa em todas as macrorregiões, exceto a Centro-Oeste (Figura 2).

**Figura 2:** Série Temporal da cobertura de óbitos de mulher em idade fértil no Sistema de Informação Hospitalar do Sistema Único de Saúde por macrorregião do país. Brasil; 2012–2020.

Na análise descritiva das características do estabelecimento associadas à cobertura de registro de óbitos de MIF a nível Brasil, verificamos que os maiores valores foram observados em estabelecimentos de maior porte (84,4% capacidade extra, 74,8% para grande porte, 69,2% para médio porte e 54,9% para pequeno porte), da esfera federal (81,6%, seguida das esferas privada, com 73,5%; estadual, com 72,2%; e municipal, com 65,5%), com maior proporção de leito adulto SUS (72,3% terceiro quartil [corte 91,5% da proporção], 74,2% de cobertura segundo quartil [corte 84,4% da proporção] e 68,1% primeiro quartil [corte 70,9% da proporção]), com atividade de ensino (universitária, 80,5%; outras modalidades de ensino 76,9% e sem atividade de ensino, 68,3%), de maior complexidade (alta 74,2%, média 64,4%, baixa 59,8%) e sem instalação física de urgência/emergência (78,2% sem instalação e 71,6% com instalação).

Na árvore de decisão, as variáveis “instalação física de urgência/emergência” e “atividade de ensino” apareceram nos primeiros nós da árvore. Foram classificados com maior probabilidade de inadequação de cobertura: hospitais com instalação física de urgência/emergência, sem atividade de ensino das esferas estadual, municipal e privada (51% da base total); e hospitais com instalação física de urgência/emergência, com atividade de ensino, de alta complexidade e com proporção de leitos adultos SUS <70% (2% da base total) (Figura 3). Na floresta aleatória, os principais preditores da cobertura de óbito de MIF foram ter instalação física de urgência/emergência, atividade de ensino, proporção de leitos adultos conveniados ao SUS, esfera do hospital e nível de complexidade (Figura 4).

**Figura 3:** Árvore de decisão para cobertura de registro de óbitos de mulher em idade fértil no SIH/SUS. Brasil, 2012–2020.

SUS: Sistema Único de Saúde; Inst. Física de urgência/emergência: existência de instalação física de urgência/emergência (sim/não); Complexidade: nível de complexidade hospitalar; Prop. de leitos adultos conv. ao SUS: proporção de leitos adultos conveniados ao SUS em relação ao total de leitos adultos existentes.

**Figura 4:** Floresta Aleatória da cobertura de registro de óbitos de Mulher em Idade Fértil no Sistema de Informação Hospitalar do Sistema Único de Saúde. Brasil; 2012–2020.

Mean Decrease Gini: parâmetro utilizado que avalia a importância das variáveis no modelo; Complexidade=nível de complexidade hospitalar (baixa, média, alta); esfera: esfera administrativa (municipal, estadual, federal, privada, não informada); Prop. de leitos adultos conv. ao SUS: proporção de leitos adultos conveniados ao SUS em relação ao total de leitos adultos existentes; atividade de ensino: universitária, outras modalidades de ensino, sem atividade de ensino; Inst. Física de urgência/emergência: existência de instalação física de urgência/emergência (sim/não).

Os dois modelos apresentaram acurácias semelhantes — em torno de 66% —, porém com performances um pouco distintas com relação às predições dos estabelecimentos com cobertura adequada ou não. A árvore de decisão apresentou maior valor preditivo positivo (82,4 *vs.* 72,4%) e especificidade (37,9 *vs*. 21,9%), enquanto a floresta aleatória apresentou maior sensibilidade (85,3 *vs.* 72,8%) e valor preditivo negativo (38,2 *vs.* 25,7%).

## DISCUSSÃO

Os resultados encontrados neste estudo mostram cobertura nacional de registro de óbito de MIF no SIH/SUS de 71,8%, com aumento no período 2012–2020. As maiores coberturas foram observadas em serviços de maior complexidade, da esfera federal, com maior proporção de leito adulto conveniado ao SUS e em hospitais de ensino; já as menores coberturas foram observadas em hospitais com instalação física de urgência/emergência.

A comparação dos nossos resultados com estudos anteriores realizados nos anos 1990 e 2000 sugere aumento da cobertura de registro de óbitos, mas esses dados devem ser analisados com cautela, devido a diferenças nos métodos utilizados. Amaral^12^, em estudo realizado em 1998, estimou uma cobertura de registro de óbito na população feminina de 52,4%, com cobertura decrescente segundo a faixa etária: 89,3% na população de 5–14 anos, 80,8% de 15–24 anos, 71% de 25–44 anos e 62,5% na população de 45–54 anos. Nesse estudo, óbitos do SIH/SUS foram comparados a óbitos no SIM que possuíam registro de assistência médica, não havendo informação referente ao serviço, se público ou privado. Essa forma de cálculo pode ter subestimado a cobertura de registros do SIH/SUS, já que o total de óbitos com assistência médica registrados no SIM incluía óbitos em serviços públicos e privados, enquanto óbitos registrados no SIH/SUS referem-se apenas às internações em serviços públicos.

Machado et al.^13^ estimaram uma cobertura de registro de óbitos em internações SUS e não SUS de 56,8% em 2009, sendo a cobertura em serviços públicos de 57,8%. Óbitos não SUS foram estimados com base no número de óbitos de beneficiários de planos de saúde divulgados pela ANS na internet, enquanto óbitos SUS foram estimados pela diferença entre o total de óbitos no SIM e os óbitos não SUS. Falhas no registro e na comunicação de óbitos em serviços privados podem ter superestimado o número de óbitos em serviços públicos no SIM e resultado numa menor cobertura de registros nesse setor.

De forma similar ao observado neste estudo, menores coberturas foram observadas por Machado et al.^13^ na região Norte e maior cobertura na região Sul. Serviços de maior complexidade, de maior porte, com atividade de ensino e com maior proporção de leitos adultos conveniados ao SUS foram os que apresentaram maior cobertura de registro de óbitos, sendo provável que a distribuição desses serviços seja desigual no país, afetando as coberturas por região. Destaca-se, entretanto, o aumento expressivo da cobertura de registro de óbito na região Norte no período avaliado, com redução da diferença em relação às outras regiões.

Maior cobertura de registro de óbitos em serviços com maior proporção de leitos SUS seria um resultado esperado, já que nem todos os óbitos ocorridos em serviços conveniados ao SUS têm financiamento público, estando registrados no SIM, mas não no SIH/SUS. Essa foi uma das explicações para a menor RMM estimada no SIH/SUS em relação ao SIM no estudo conduzido por Ranzani et al.^10^. Já a maior cobertura em serviços de maior porte, de maior complexidade e com atividade de ensino pode refletir uma melhor estrutura e organização dos sistemas de registro e documentação médica em estabelecimentos com essas características. Maior cobertura de registro de internações em hospitais de maior porte já havia sido relatada por Machado et al.^13^, bem como melhor desempenho da gestão hospitalar em hospitais de maior porte e complexidade^26^.

O fator que mais afetou a cobertura de registro de óbitos foi a existência de instalação física de urgência/emergência no hospital. Menor registro de óbitos em hospitais com serviços de urgência/emergência já havia sido relatado previamente em estudo que avaliou mortalidade por infarto agudo do miocárdio^5^. Nossa hipótese para esse resultado é de que nesses hospitais é feita a emissão de declaração de óbito para pacientes admitidos na urgência/emergência que evoluíram para óbito, sem a correspondente emissão de AIH para esses mesmos indivíduos. Dessa forma, óbitos são registrados no SIM, mas não no SIH/SUS, resultando em menor cobertura. O sub-registro de óbitos de MIF em serviços de urgência/emergência pode afetar as estimativas de óbito materno, principalmente aqueles ocorridos no puerpério, por complicações não específicas da gestação, tais como infecções.

Os modelos de predição apresentaram acurácia semelhante, diferindo nas medidas específicas, com maior sensibilidade e valor preditivo negativo na floresta aleatória. A floresta aleatória costuma gerar melhores predições que a árvore de decisão, mas o resultado da árvore de decisão pode ser de mais simples implementação pelos gestores, uma vez que gera pontos de corte para variáveis (quantitativa ou qualitativa) permitindo a visualização de “regras de classificação” e respectivos valores. Por exemplo, na árvore apresentada, hospitais com instalação física de urgência/emergência; sem atividade de ensino; das esferas estadual, municipal e privada apresentaram a maior probabilidade de cobertura inadequada, representando 50% da base, devendo ser o foco prioritário de estratégias de melhoria.

Este estudo apresenta limitações relacionadas à qualidade do registro de dados. Foram excluídas do SIM as DO com CNES não preenchido ou inválido, bem como DO e AIH com número de CNES sem correspondência no SIH/SUS e no SIM. Unidades hospitalares que apresentavam cobertura superior a 100% também foram excluídas, pela possibilidade de erro de registro, já que o SIM apresenta cobertura superior a 90% no país, e coberturas no SIH/SUS superiores a 100% não seriam esperadas. Esses problemas não ocorreram de forma homogênea nos estados, podendo ter afetado as coberturas por UF, além da cobertura nacional. Erros de registro do CNES e a emissão de AIH para internações não realizadas são possíveis explicações para uma cobertura no SIH/SUS superior a 100%.

Foram comparadas as frequências de registro de óbito do SIM e SIH/SUS em cada unidade hospitalar, não sendo possível afirmar que haja concordância entre os óbitos registrados. É possível que exista sub-registro da informação do óbito, já que o desfecho da internação não afeta o pagamento dos procedimentos realizados^13^. Por outro lado, erros de digitação podem acarretar sobre-registro de óbitos, já que não existem críticas no sistema e a digitação da CID do óbito não é obrigatória no SIH/SUS. Também não foram avaliadas as causas de óbito registradas no SIH/SUS e sua concordância com as causas registradas no SIM.

Bases de dados administrativos têm por objetivo o pagamento da assistência prestada, e não a avaliação da qualidade do cuidado, podendo apresentar falhas de preenchimento de variáveis relevantes^27,28^. Estudos realizados em outros países também apontam fragilidades na disponibilidade, acessibilidade e qualidade da informação sobre óbitos nos sistemas de registro hospitalar^29,30,31^ e a necessidade de capacitação em processamento de dados, incluindo armazenamento e revisão periódica de prontuários^30^.

Como conclusão, o SIH/SUS apresentou cobertura elevada de registro de óbitos de MIF em todo o país, sendo uma fonte importante de informação para a vigilância da morbimortalidade materna no país. Estratégias devem ser adotadas visando melhoria no registro dos dados nos diversos sistemas de informação hospitalares, incluindo óbitos no SIH/SUS. Estudos futuros devem avaliar a concordância do registro de óbitos no SIM e no SIH/SUS, bem como de suas causas.
